# Impaired multiple object tracking in children with chromosome 22q11.2 deletion syndrome

**DOI:** 10.1186/1866-1955-4-6

**Published:** 2012-04-12

**Authors:** Margarita H Cabaral, Elliott A Beaton, Joel Stoddard, Tony J Simon

**Affiliations:** 1 Department of Psychiatry and Behavioral Sciences and the Medical Investigation of Neurodevelopmental Disorders (MIND) Institute, University of California Davis Medical Center, 2825 50th Street, Sacramento, CA 95817, USA

**Keywords:** Attention, Children, Chromosome 22q11.2 deletion syndrome (22q11.2DS), DiGeorge Syndrome, Multiple object tracking, Spatiotemporal attention, Velocardiofacial syndrome (VCFS)

## Abstract

**Background:**

Chromosome 22q11.2 Deletion Syndrome (22q11.2DS) occurs in approximately 1:4,000 live births with a complex and variable presentation that includes medical, socioemotional and psychological symptoms with intellectual impairment. Cognitive impairments in spatiotemporal and visuospatial attention have also been reported. However, maintenance of selective attention to dynamic and interacting objects has not been systematically investigated in children with 22q11.2DS.

**Methods:**

We used a multiple object tracking task to assay capacity and resolution performance of children with 22q11.2DS aged 7 to 14 years versus age-matched typically developing (TD) peers.

**Results:**

Children with 22q11.2DS but not TD children demonstrated impaired performance when task demands increased due to an increase in the number of targets presented, but not from an increase in object speed. Task performance in children with 22q11.2DS was also unrelated to intelligence or measures of attention deficit hyperactivity disorder.

**Conclusions:**

These findings suggest that children with 22q11.2DS may be particularly susceptible to dynamic crowding of objects with increasing cognitive demands related to monitoring multiple targets reflecting a reduced acuity in spatiotemporal cognitive representation.

## Background

Chromosome 22q11.2 deletion syndrome (22q11.2DS), also known as DiGeorge [1], velocardiofacial VCFS; [2] and conotruncal anomaly face [3] syndromes among other labels, results from a hemi-zygotic interstitial deletion between 1.5 and 3 Mb on the q11 band of chromosome 22. It is the most common survivable chromosomal micro-deletion with a prevalence of approximately 1:4,000 live births [4-6]. Syndrome presentation is highly variable, but physical dysmorphisms [7], socioemotional difficulties [8] and cognitive impairments in both the verbal and non-verbal domains [9,10] are characteristic of this population.

Cognitive deficits commonly reported with 22q11.2DS include difficulties with numerical thinking [11,12] that may arise from decreased representational resolution for both space and time [13-15], which Simon [15] labels 'spatiotemporal hypergranularity' (see also [16]). As a result, children with 22q11.2DS may have greater difficulty attending to multiple objects moving and interacting dynamically in visual space. Reduced acuity in spatiotemporal representation increases apparent crowding between interacting objects, thereby reducing accessibility to individual items [17]. Successful interaction with and navigation in dynamic visual environments also requires rapid, accurate and continuous shifting of attention to changes in the visual field. Thus, the potential for crowding is exacerbated by unpredictable motion, further influencing the capacity of items that can be tracked.

Multiple object tracking (MOT) tasks have been used as a means to assess the capacity and resolution of spatiotemporal attention [18-20]. In a MOT task, study participants are asked to monitor the changing positions of several target objects in a field of identical objects that serve as distractors. This requires maintenance of attention over both space and time. Task performance is modulated by several parameters, including the number of objects in the field, the size of the field, the speed of which the objects move, and the length of the tracking period.

In the current study, we use a MOT task on a computerized touch screen based on Trick and colleagues' (2005) "catch the spies" MOT task developed for children to investigate the ability of children with 22q11.2DS to accurately maintain attention to dynamically interacting targets and distractors in visual space over time. We hypothesized that when asked to dynamically track one or more targets in a field of moving distractors, children with 22q11.2DS would demonstrate increasingly impaired performance with increasing task demands (that is, more targets and/or faster motion) than typically-developing (TD) age-matched peers.

## Methods

### Participants

Participants were tested at the University of California Davis (UC Davis) Medical Center M.I.N.D. Institute in accordance with a UC Davis Institutional Review Board approved protocol. Parental consent and child assent were obtained for all participants. Diagnosis of 22q11.2DS was confirmed in children using fluorescent *in-situ *hybridization analysis. Typically developing (TD) child participants had no known diagnosis of developmental disorder. Inclusion criteria for study recruitment in both groups included: proficiency in English, no brain infarct, central nervous system infection, head injury or other focal neurological abnormality, and as part of a larger study - no issues contraindicated for MRI. All participants had normal or corrected-to-normal vision.

Intelligence quotient (IQ) measures were obtained using the Wechsler Intelligence Scale for Children, Version 4 WISC-IV; [21] and the Wechsler Abbreviated Scale of Intelligence WASI; [22]. One child with and seven without 22q11.2DS were administered the WASI. Attention deficit/hyperactivity disorder (ADHD) behavior was assessed in children with 22q11.2DS using the parent-report Swanson, Nolan, and Pelham version IV (SNAP-IV) rating scale [23].

A total of 47 children with 22q11.2DS and 45 TD children were recruited for the study. Participants were subsequently excluded from further analyses if they were unable to complete both MOT tasks (described below) and/or if their performance on either task was below chance or 2.5 SD below the mean group performance. In total, 34 children with 22q11.2DS (n = 16 male and 18 female, M age = 10 years, 11 months, SD = 2 years, 2 months) and 41 TD children (n = 22 male and 19 female, M age = 10 years, 2 months, SD = 2 years, 3 months) were able to complete both the 30 frames per second (fps) and 60 fps tasks and were included in further analyses.

As a group, children with 22q11.2DS had lower Full Scale scores (22q11.2DS: M = 77.52, SD = 10.99 vs. TD: M = 111.59, SD = 11.98) (t(68) = 12.35, *P *= 0.0001); Processing Speed Scale scores (22q11.2DS: M = 79.38, SD = 10.90 vs. TD: M = 108.43, SD = 14.80) (t(60) = 8.84, *P *= 0.0001); Working Memory Scale scores (22q11.2DS: M = 83.32, SD = 14.00 vs. TD: M = 103.90, SD = 12.42) (t(59) = 6.07, *P *= 0.0001); Verbal Comprehension Scale scores (22q11.2DS: M = 82.48, SD = 11.43 vs. TD: M = 110.00, SD = 14.42) (t(67) = 8.73, *P *= 0.0001); and Perceptual Reasoning Scale scores (22q11.2DS: M =, SD = vs. TD: M =, SD =) (t(67) = 9.14, *P *= 0.0001), as measured by the WISC-4 or the WASI. Groups did not differ in mean age (t(73) = 1.48, *P *= 0.14)) or gender composition (χ^2 ^= 0.32, *P *= 0.65) and excluded and included children did not differ in Full Scale IQ, age, gender composition, or SNAP-IV measures of inattentive-, hyperactive/impulsive-, or combined-type ADHD (all *P*s > 0.2).

### Multiple object tracking task

**Figure 1:**
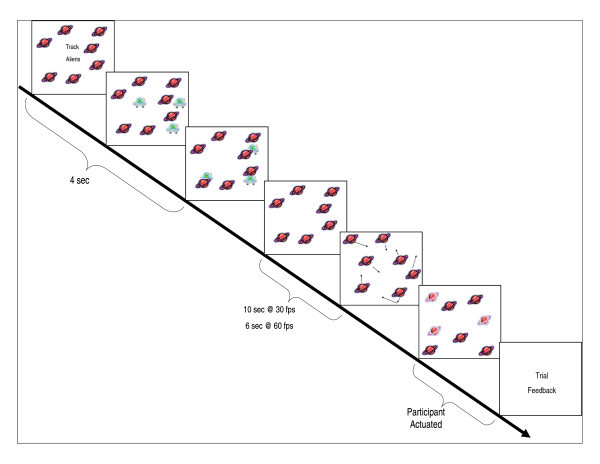
**Schematic illustration of an example trial in the multiple object tracking task**.

Children were trained on a simple two-part version of the task and were told that they were helping space explorers find friendly aliens hiding on one or more of the planets they were exploring. A schematic of an example three-target trial is illustrated in Figure 1. In training, children observed screen-captured images illustrating each part of the MOT task in order. Children controlled the amount of time that they observed each image before advancing to the next image. When they reported and demonstrated that they understood the task requirements they proceeded to engage the interactive MOT task.

Stimuli were presented using Macromedia Projector (Adobe, San Jose, CA, USA), running on Windows XP operating system (Microsoft, Redmond, WA, USA) on a touch-sensitive monitor (ēlo Touchsystems Intellitouch v4.40, Menlo Park, CA, USA) placed 60 cm away from the participant. Measurement of the black tracking field was 756 by 756 pixels. Each trial began with seven identical cartoon planets measuring 56.7 by 56.7 pixels surrounding the instructions "Track Aliens", centered at fixation. Next, one, two or three identical cartoon aliens measuring 94.5 by 94.5 pixels appeared and then shrank to 56.7 by 56.7 pixels before moving 'behind' a planet to 'hide'. To signal the start of motion, the planets with 'hidden' aliens flashed for one second then moved randomly and independently of one another at three pixels per frame in vertical, diagonal and horizontal directions. Objects collided with one another or the field boundary but did not overlap or cross over the field boundary. Objects changed directions when colliding with one another or the field boundary, independently changing direction with a 0.005 probability per frame. The task was presented in two parts at an apparent frame rate of 30 fps (8.25 ms/frame) and 60 fps (16.5 ms/frame) for all participants. Total tracking time for the 30 fps version was 10 sec and 6 sec for the 60 fps version. Length of tracking time was reduced in the 60 fps version of the task because pilot data demonstrated that several children with 22q11.2DS were performing at less than chance levels when required to track targets at 60 fps for 10 sec. Both the 30 and 60 fps versions of the task were made up of 30 trials: 10 with one target, 11 with two targets, and 9 with three targets presented pseudo-randomly. The 30 and 60 fps versions were presented in random order.

Participants were instructed to visually track the target objects, identifying them by pressing them on the monitor using their dominant index finger. Selected planets became 'highlighted' until the participant ended the trial by pressing the spacebar button on the computer keyboard. Trials were not time limited and participants were able to deselect highlighted planets and select other choices as often as they wished.

### Calculation of corrected task performance score

Based on Hulleman (2005) and O'Hearn *et al. *(2010), we calculated performance corrected for error using the following formula to generate a *k*-score for each trial in the two separate tasks (Hulleman, 2005; O'Hearn *et al. *2010):

$$k=(\mathrm{nc}\text{ - }t^{\wedge }2)/(n+c\text{ - }2t)$$

where *n *equals the number of distractor elements (seven planets) that appear on the screen at the beginning of each trial, *t *equals the number of targets (one, two or three aliens) to track, with *c *equal to the number of correct choices made by the participant during the response phase of a given trial. The *k*-scores generated for each trial were averaged within conditions to generate a one-target, two-target and three-target mean *k*-score for each participant. These values accounted for the relative difficulty of the trials with regards to the number of targets and distractors. Perfect accuracy on all trials would generate a *k*-score of 1, 2, and 3 for the one-target, two-target and three-target conditions respectively.

Next, we generated a proportional performance score by dividing participants' k-scores by the highest possible k-score value for each condition. Statistical analyses were performed on proportional *k*-scores. Data for children whose performance was still below chance level or 2.5 SD below group mean performance after this correction was removed from further analyses.

### Error calculation

If the participant selected a target more than once, it was included as a final choice if the number of selections was an odd number, given that an even number of selections implies that for every selection a corresponding de-selection of the same target followed. Final choices for each trial were characterized as the first, second and third non-identical selections depending on the number of targets in the trial (for example, one, two or three targets). The number of extra non-identical selections was calculated as 'Intrusion errors'.

## Results

All analyses were conducted using SPSS version 20 (IBM Corporation, Armonk, NY, USA) with an accepted confidence level of 0.95 (alpha = 0.05) reporting exact statistics or using Bonferroni correction for multiple comparisons. Group differences in task performance were measured by comparing proportional k-scores across GROUP (22q11.2DS, TD) and OBJECT SPEED (30 fps, 60 fps) and NUMBER OF TARGETS (1 TARG, 2 TARG, 3 TARG) controlling for AGE and GENDER using a repeated measures multivariate analysis of variance (MANOVA). There was a main effect of GROUP (*F*(1, 70) = 18.48, *P *< 0.0001, η2 = 0.21) but not of AGE (*F*(1, 70) = 2.65, *P *= 0.11) or GENDER (*F*(1, 70) = 0.35, *P *= 0.56). An interaction between GROUP and NUMBER OF TARGETS (*F*(2, 140) = 12.39, *P *< 0.0001, η2 = 0.15) also reached statistical significance. There were no other statistically significant main effects or interactions for OBJECT SPEED, NUMBER OF TARGETS, AGE, or GENDER (all *P*s > 0.37).

Next, we used Repeated Measures MANOVA with the AGE and GENDER covariates removed to explore the GROUP by NUMBER OF TARGETS interaction at 30 fps and then at 60 fps. At 30 fps, there was a significant main effect of GROUP (*F*(1, 73) = 10.51 *P *= 0.002, η2 = 0.13). Within groups at 30 fps, there was a significant main effect of NUMBER OF TARGETS (F(2,146) = 9.63, *P *< 0.001, η2 = 0.117) and an significant interaction of GROUP and NUMBER OF TARGETS (F(2,146) = 4.07, *P *= 0.02, η2 = 0.05).

At 60 fps there was a significant main effect of GROUP (F(1,73) = 8.83, *P *= 0.004, η2 = 0.11). Within groups at 60 fps, there was a significant main effect of NUMBER OF TARGETS (F(2,146) = 13.36, *P *< 0.0001, η2 = 0.16) and an significant interaction of GROUP and NUMBER OF TARGETS (F(2,146) = 6.62, *P *= 0.002, η2 = 0.09).

**Figure 2:**
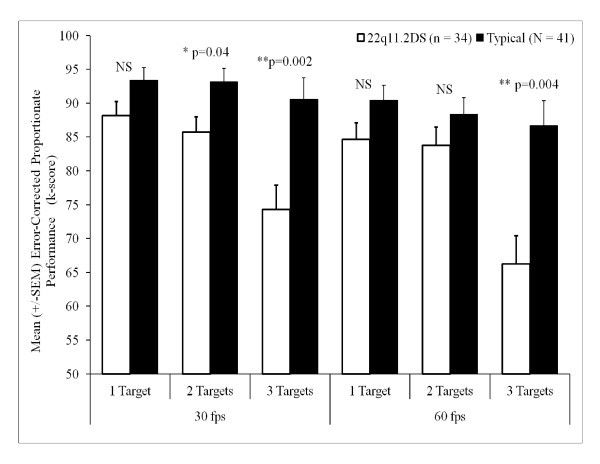
**Proportional performance of target conditions at 30 and 60 fps between TD and 22q11.2DS groups**.

Group differences in performance were then interrogated at each level of NUMBER OF TARGETS with pairwise comparisons corrected for multiple comparisons using the Bonferroni method. As illustrated in Figure 2, the 22q11.2DS group demonstrated a statistically significant impairment in performance compared with TD children at 30 fps when tracking two targets (F(1,73) = 4.55, *P *= 0.04, η2 = 0.059) at 30 fps and 3 targets at 30 fps (F(1,73) = 10.89, *P *= 0.002, η2 = 0.13) or 60 fps (F(1,73) = 8.82, *P *= 0.004, η2 = 0.11). Group differences indicating poorer performance by the 22q11.2DS group versus the TD group approached but did not reach statistical thresholds in performance when tracking one target at 30 fps (F(1,73) = 2.80, *P *= 0.10) or 60 fps (F(1,73) = 2.97, *P *= 0.09) or when tracking two targets at 60 fps (F(1,73) = 1.33, *P *= 0.25).

**Figure 3:**
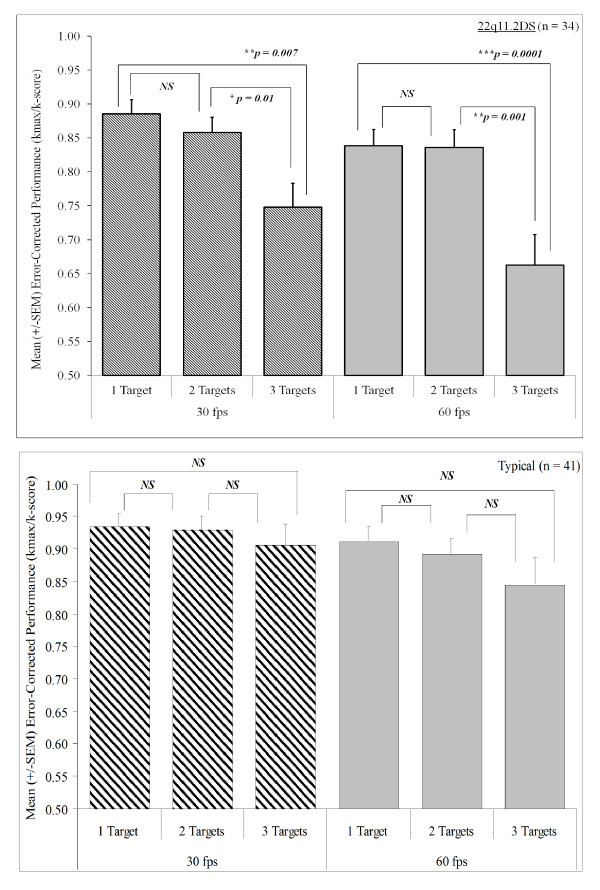
**Within group proportional performance of target versus speed for (a) 22q11.2DS and (b) TD children**.

*Post-hoc *analyses of NUMBER OF TARGETS were conducted using Bonferroni-corrected paired t-tests at each level of OBJECT SPEED within groups. As illustrated in Figure 3a, b, within the 22q11.2DS group in the 30 fps condition, there was no significant difference in error-corrected performance between the one- and two-target tasks (*t*(33) = 1.05, *P *= 0.30) but a significant performance impairment difference between the one- and three-target tasks (*t*(33) = 2.89, *P *= 0.007) and between the two- and three-target tasks (*t*(33) = 2.72, *P *= 0.01). In the 60 fps condition, performance did not differ for the 22q11.2DS group between the one- and two-target conditions (*t*(33) = 0.51, *P *= 0.96) but performance was lower on the three-target task than the one-target (*t*(33) = 3.90, *P *= 0.0001) or two-target tasks (*t*(33) = 3.75, *P *= 0.001). There were no significant differences in task performance when tracking one, two or three targets in TD children for either the 30 or 60 fps version of the task (all *P*s > 0.1).

Although children with 22q11.2DS had significantly lower FSIQ (*P *= 0.001), compared to TD children, FSIQ did not predict performance on any component of the MOT task (all *P*s > 0.27) in children with 22q11.2DS. However, within the TD group, FSIQ was positively correlated with performance on one-target 30 fps trials (r(37) = 0.33, *P *< 0.05] but not for other trial types. SNAP-IV scores of inattentive, hyperactive/impulsive and combined-type ADHD for children with 22q11.2DS were treated as continuous variables and correlated against MOT task performance. SNAP-IV scores did not predict performance on any component of the MOT task (all *P*s > 0.20).

## Discussion

For children with 22q11.2DS, having two or more targets to track negatively impacted MOT task performance. Increasing the speed of object motion from 30 to 60 fps did not have a statistically measurable effect on performance in either group. In contrast to the 22q11.2DS group, MOT task performance in the TD group was unaffected by the number of targets or speed of motion. MOT task performance in the 22q11.2DS group was not predicted by intelligence measures or ADHD symptoms. Unpacking inter-group performance by target number, we found that tracking a single target was statistically the same for both groups at the 30 fps object speed. Adding a second target at 30 fps, the 22q11.2DS group showed impaired performance relative to their TD counterparts that was not apparent between the groups when tracking two targets at 60 fps. This suggests that increasing speed in combination with the additional target began to affect children in the TD group at 60 fps leveling group performance to that of children with 22q11.2DS. This finding also indicates that speed-induced dynamic crowding is not specific to children with 22q11.2DS and that increasing the object speed was increasing task demands in part even though a significant main effect of speed was not statistically detectable. As task demands further increased with three targets at 30 and 60 fps, children with 22q11.2DS demonstrated a significant drop in performance and accuracy that TD children did not experience. This suggests that capacity to track objects in space and time is impaired in children with 22q11.2DS.

The origins of this impairment in children with 22q11.2DS may stem from reduced acuity in spatiotemporal representation. Increasing the number of targets serves to increase the number of object interactions and the likelihood that attention will erroneously shift from the target object to a distractor. Poorer acuity in spatiotemporal representation of moving objects in space increases apparent crowding and interaction of objects. In the brain, the origin of these impairments in children with 22q11.2DS may stem from atypical brain development in frontoparietal cortical circuitry on which attention depends [24,25]. However, performance on the MOT task does not stem from a global developmental, intellectual, or visual processing impairment. Children with 22q11.2DS do not differ from TD children in terms of their response time to a single, but unpredictably appearing visual stimulus [15] and measures of IQ did not predict performance of children with 22q11.2DS in the present study.

A variety of factors affect MOT task performance. For example, in TD adults, tracking four or more targets begins to impact MOT performance and accuracy [19,20]. Our model was a child-friendly version consisting of three targets, which is the difficulty level where typically developing 6- to 12-year-olds was shown to differ noticeably [26]. Non-optimal performance even for the TD group shows that this adaptation to using a maximum of three targets was appropriate. Further, increasing the number of targets presented negatively impacted 22q11.2DS performance to a greater degree than TD performance.

Although there was no statistically measurable difference in performance from increasing object speed from 30 to 60 fps in the present study, speed has been shown to modulate spatial attention capacity. Alvarez and Franconeri [18] report that decreasing object motion speed with increasing numbers of objects maintains accurate tracking capacity suggesting a division of attention amongst targets. Greater rates of object interaction in combination with greater apparent object crowding results in poorer object discrimination [27] and more opportunities to erroneously shift attention from the target object to a distractor. We expect that increasing object speeds beyond 60 fps would likely have a measurable effect on performance [18] for children both with and without 22q11.2DS.

Although the TD group generally outperformed the 22q11.2DS group, errors made by TD children and the positive correlation between FSIQ and performance on the one-target, 30 fps trials but not for the one-target trials at 60 fps for TD children are interesting. The presentation of target number trials is random and it might be expected that the performance would decrease with increased task loads in children with lower FSIQ. It is possible that children with lower FSIQ are deploying different cognitive strategies across trial types. Capacity and resolution of spatiotemporal attention likely continues to develop beyond the age range designated in this study, which is consistent with the findings of Trick and colleagues [26]. Prospective studies could reveal that developmental delay in children with 22q11.2DS contributes to performance impairments, and that with time they may perform at a level similar to TD children.

Although the findings demonstrate impaired attentional capacity for tracking multiple objects over time in children with 22q11.2DS, the study is not without limitations. The length of the trials was adjusted so that the 30 fps trials were 4 sec longer than the 60 fps trials. Preliminary data collection revealed that several children with 22q11.2DS were performing at less than chance when attempting the 10 sec duration 60 fps trials. A ceiling effect was not evident in the TD children but larger performance impairment may have been evident in the TD group had the 10 sec 60 fps condition been used. Although, intelligence was not predictive of MOT task performance in children with 22q11.2DS, the study could have also benefited from the inclusion of a second control group comprised of age-matched children with another neurodevelopmental disorder. This would serve to test the specificity of hypothesized spatiotemporal and attentional impairment in children with 22q11.2DS. The study could also be improved by the addition of eye-tracking technology. Individual differences in ability to saccade and maintain gaze on a moving target could explain some performance differences. Eye tracking could also be used to measure specific incidences where gaze and attention shifts from the targets to distractors.

## Conclusions

Unrelated to intellectual ability, increasing MOT task demands in the form of additional targets negatively impacted performance in children with 22q11.2DS but not TD children. Children with 22q11.2DS may be particularly susceptible to spatial interactions and dynamic crowding of moving objects because of a reduced acuity in spatiotemporal resolution and representation.

## Abbreviations

22q11.2DS: Chromosome 22q11.2 deletion syndrome; ADHD: Attention deficit hyperactivity disorder; Fps: Frames per second; MANOVA: Multivariate analysis of variance; MOT: Multiple object tracking; SNAP-IV: Swanson, Nolan, and Pelham version IV; TD: Typically developing; VCFS: Velocardiofacial syndrome

## Competing interests

All authors state that there are no actual or potential conflicts of interest, including financial, personal or any other relationships or organizations within three years of beginning this work, that could inappropriately influence, or be perceived to influence, their work.

## Authors' contributions

MHC acquired the data as well as played a primary role in data collection, analysis and interpretation in addition to drafting the manuscript. EAB played a critical role in generating the statistical models for analysis, provided data interpretation and contributed significantly to drafting the manuscript. JS participated in the design of the study and provided data interpretation and support. TJS conceptualized the study and played a primary role in the task design and data interpretation. All authors have read and approved the final manuscript.
